# Protocol for Characterization of Addiction and Dual Disorders: Effectiveness of Coadjuvant Chronotherapy in Patients with Partial Response

**DOI:** 10.3390/jcm11071846

**Published:** 2022-03-26

**Authors:** Ana Adan, José Francisco Navarro, on behalf of ADDISCHRONO Group

**Affiliations:** 1 Department of Clinical Psychology and Psychobiology, School of Psychology, University of Barcelona, Passeig de la Vall d’Hebrón 171, 08035 Barcelona, Spain; 2 Institute of Neurosciences, University of Barcelona, 08035 Barcelona, Spain; 3 Department of Psychobiology, School of Psychology, University of Málaga, Campus de Teatinos s/n, 29071 Málaga, Spain; navahuma@uma.es

**Keywords:** substance use disorder, dual disorders, major depressive disorder, schizophrenia, circadian rhythm, genetic polymorphisms, neurocognition, chronobiological therapy, exposure to natural light, clinical course

## Abstract

This protocol aims to characterize patients with dual disorders (DD; comorbid major depression and schizophrenia) compared with patients with only a diagnosis of substance use disorder (SUD) and those with only a diagnosis of severe mental illness (SMI; major depression and schizophrenia), evaluating clinical and personality characteristics, circadian rhythmic functioning, genetic polymorphism and neuropsychological performance in order to obtain a clinical endophenotype of differential vulnerability for these diagnostic entities. Patients will be divided into three groups: DD (45 men with comorbid schizophrenia, 45 men and 30 women with major depression), SUD (*n* = 90, with a minimum of 30 women) and SMI males (45 with schizophrenia, 45 with major depression). All patients will be under treatment, with at least three months of SUD abstinence and/or with SMI in remission or with stabilized symptoms. Outpatients of both sexes with insufficient restoration of circadian rhythmicity with SUD (*n* = 30) and dual depression (*n* = 30) will be asked to participate in a second two-month study, being alternately assigned to the condition of the chronobiological adjuvant approach to the treatment of regular hour habits and exposure to light or to the usual treatment (control). The effect of the intervention and patient compliance will be monitored with a Kronowise KW6^®^ ambulatory device during the first two weeks of treatment and again at weeks 4 and 8 weeks. After completing the evaluation, follow-up of the clinical evolution will be carried out at 3, 6 and 12 months. This project will allow us to analyze the functional impact of DD comorbidity and to develop the first study of chronobiological therapy in the treatment of SUD and dual depression, with results transferable to the clinical setting with cost-effective recommendations for a personalized approach.

## 1. Introduction

Substance use disorder (SUD) affects millions of people worldwide with possible devastating personal consequences, which require a specific, intense and long-term therapeutic approach to recovery. This disorder generates a high economic cost and very significant drop-out and relapse rates. Dual disorders (DD), defined as the coexistence of an SUD and a severe mental illness (SMI) not secondary to the first, have become increasingly prevalent in recent years [1]. Among the comorbidities of SUD, diagnoses of major depression and schizophrenia are the most frequent. In addition, about 50% of schizophrenic patients and 32% of patients with affective disorders also have an SUD, excluding nicotine and caffeine [2,3].

In both SUD and DD, the male sex is most prevalent in the clinical setting (around 80%), with cocaine or alcohol being the substances that produce the most frequent dependence in Spain and other European countries, followed by cannabis [4,5], although most patients develop a pattern of polyconsumption. Currently, DD has an enormous clinical impact due to the difficulty in diagnosis and therapeutic management, as well as its high healthcare cost. Thus, it has been demonstrated that DD patients are more likely to exhibit an increase in symptoms, more relapses, hospitalizations, medical illnesses and suicidal risk, as well as greater victimization, social isolation and premature death [6,7]. Patients with DD also tend to show poor adherence, worse treatment response and lower quality of life [8,9] as compared to those with a single pathological condition.

### 1.1. Circadian Rhythmicity

The alteration of the circadian rhythmic system (amplitude reduction, phase delay, lower interdaily stability, worse sleep quality and wakefulness) has been considered a possible marker for SUDs [10], major depression [11,12] and schizophrenia [13,14]. In young people with affective disorders in early stages, alterations are related to social and occupational functioning [15], whereas in adolescents at risk of developing psychosis, they are related to the severity of psychotic symptoms and the deterioration of social functioning at one year of follow-up [14]. In the middle and advanced phases, circadian involvement may meet chronodisruption criteria and is correlated with symptomatic severity, more remission difficulty, worse prognosis and worse quality of life in patients [16]. However, research has focused on the presence of sleep disorders in SUD (with 70% of patients who come to treatment), major depression and schizophrenia [17] and, to a lesser extent, in the rhythms of circadian markers, such as body temperature.

Numerous review studies have proposed, from the observations of circadian dysregulation in SUD patients prior to treatment and during detoxification, that it would be useful to incorporate chronobiological strategies both in the therapeutic approach [17,18] and in prevention [19], similar to how these strategies have been applied in patients with seasonal or non-seasonal depression. A lower contrast between day and night (reduced amplitude) and a greater fragmentation of the rhythm and fragility of the sleep–wake cycle have been considered poor indicators of circadian rhythmicity, which may reflect an immaturity of the circadian system [20]. Our group has demonstrated that patients in treatment with only SUD [21] and, to a greater extent, with dual depression [22] exhibit a lower percentage of rhythm, interdaily stability and amplitude due to less adequate diurnal values related to onset age of consumption, severity of addiction, withdrawal time and type of treatment (residential/outpatient). These observations suggest a slower restoration of the homeostatic process (S), and the need for sleep during wakefulness compared to the circadian process (C) of the revised Borbély model [23].

Despite the increasing interest and the possible clinical utility attributed to chronobiology for the understanding and management of SUD and DD, studies with humans, although offering very promising data, are very limited. The development of ambulatory devices for the evaluation of a set of objective parameters of the functioning of the circadian system, such as the Kronowise KW6^®^ (Kronohealth, Murcia, Spain) (distal body temperature, activity, intensity and type of environmental light), offers enormous possibilities to advance knowledge of variables key to clinical practice. Body temperature is considered a good estimator of circadian endogenous rhythmic expression, whereas exposure to light is valued as an indicator of synchronization with the solar cycle and of activity expressing the circadian behavioral habits of individuals. Its use in this investigation may result in obtaining a clinical marker of response to treatment and risk of relapse, with very affordable technology and costs and with good acceptance by patients.

### 1.2. Genetic Polymorphisms and Clock Genes

Molecular genetics plays an important role in the identification of new risk factors and pathophysiological mechanisms for the vast majority of neuropsychiatric disorders, which are useful for the development of new approaches for both diagnosis and treatment. Single nucleotide polymorphisms (SNPs) represent the most frequently studied type of genetic variation in human molecular genomics. Currently, the human SNP database includes more than 30 million of SNPs (http://www.ncbi.nlm.nih.gov/SNP; accessed on 21 March 2022). A fraction of SNPs has direct functional effects and is the basis for a large number of interindividual differences, as well as being involved in the predisposition to diseases and endophenotypes related to the individual response to drugs, among many others [24].

Among the circadian or clock genes, the Period genes (*PER2* and *PER3*) stand out for their implications, encoding proteins that are increased during the night hours and decreased during daytime. These inform the NSQ cells and peripheral organs as to what time it is approximately. There is a complex interaction between the clock genes and the functioning of the organism, with a bidirectional relationship between circadian rhythmic expression and various mental disorders. Thus, certain characteristics of circadian rhythmicity (phase delay and reduced amplitude, among others) influence the risk of developing an SUD, and this, in turn, is a factor that impairs proper circadian rhythmicity by modifying the gene expression of clock genes [17]. Genetic studies support the thesis that circadian genes are directly involved in the regulation of the dopaminergic reward circuit and that in vulnerable individuals, alterations of the circadian system could contribute to modifying the value of the reward and the motivation for substance use. In this sense, neuroimaging studies show altered neuronal responses towards the reward in evening subjects [19].

In humans, the *PER2* gene has the greatest influence on NSQ and is only expressed in the CNS. Polymorphisms in this gene have been associated with compulsive stress-mediated alcohol consumption, in addition to being involved in the expression of period and phase. Research with animal models of addiction has shown that a reduced expression of Per2 is related to a decreased production of the MAOA enzyme, as well as to increases in dopamine and improvements in depressive symptomatology [12,25], whereas D2R activity contributes to reducing the expression and rhythm of Per2 in the reinforcement system (striatum) [18]. Thus, serotonin at adequate levels regulates the circadian expression of Per2 (low during the day and elevated at night) [25]. The *PER3* gene, the most robust of the rhythmic genes, has been related to the circadian typology phase in response to morning light exposure and to differences in cognitive impact (executive functions and memory) [26]. *PER3* polymorphisms and levels of their gene expression have also been associated with addiction, schizophrenia and major depression, as well as response to SSRI-type antidepressants [12,26].

*PER2* (rs934945) has been found, in Latin American participants, to be associated with morning alertness and activity planning, whereas *PER3* (rs2640909) is associated with morningness–eveningness (phase) [27]. Therefore, we intend to further analyze this aspect in the present investigation. We are not aware of any study exploring the presence of these polymorphisms in patients with SUD or DD. Our interest in this work, being aware of the small number of participants regarding the current genetic research in consortium and with global databases, is to try to establish their possible relationships with the other variables under study.

In relation to other genetic polymorphisms, there are numerous studies in patients diagnosed with SUD, major depression or schizophrenia, whereas in DD, knowledge is very limited, with heterogeneous data. Based on the diagnoses and variables considered in the present study, we focused our interest on the exploration of the *BDNF* (brain-derived neurotrophic factor; rs6265), *APOE* rs429358, rs7412 (E2, E3 and E4) and *MAOA* (uVNTR) genes, considering their implications for cognitive performance and circadian rhythmic expression, as well as the possible relationship with the response to the proposed chronobiological intervention.

BDNF rs6265 (Val66Met) is one of the main candidate genes in major depression [28] and in schizophrenia [29], and it has been implicated in cognitive deficits exhibited by the patients. Likewise, the existence of *BDNF* polymorphisms has been confirmed in DD patients with schizophrenia [30] and with altered cognitive performance. The data on *APOE*, which acts as a regulator of several mechanisms of cerebral plasticity, are controversial in schizophrenia, with both positive and negative conclusions but with some involvement as a protective factor in Asian populations [31]. In healthy individuals, a polymorphism in the promoter region of the *MAOA* gene has been related to the quality of wakefulness [32], whereas polymorphisms in *MAOA* have been associated with major depression and with suicidal behavior in men [33].

### 1.3. Cognitive Performance

Neuropsychological impairment associated with SUD, both psychotic and depressive, is a field of study with numerous published papers [34] (for review). Different cognitive deficits have also been identified in SUD, associated with problems of behavioral inhibition, decision making, sustained attention and strategy planning [35,36]. In all cases, there is evidence that deficits do not always recover after the remission of the disorder and that cognitive rehabilitation and psychosocial interventions in the therapeutic approach are key factors for recovery.

The data collected in patients with DD are scarce and heterogeneous, although—to a greater or lesser extent—deficits in attention, memory and executive functions are consistently observed, in agreement with neurochemical and neurofunctional affectations. These deficits have been described both in relation to schizophrenia [37,38], with deficits in flexibility and inhibition in executive functions, and affective disorders/major depression, in which the magnitude of the deficits is comparable to that observed in SUD [39]. Various studies carried out by our group suggest that a complete neuropsychological evaluation is necessary, although compatible with the pressure of clinical practice, and that the affectation is modulated by factors such as age, the age at onset of SUD, the main type of dependence drug and premorbid Intellectual Quotient (IQ) in both SUD [40] and dual schizophrenia [41,42,43]. That is, in dual schizophrenia, there is a complex model in which young patients have less vulnerability than those with only schizophrenia due to the presence of neurocognitive deficits, regardless of the domain studied, although these deficits become evident around age 50, associated with the risk of neurodegeneration and the main type of drug consumed. In relation to decision making, this is less appropriate in patients with dual schizophrenia regarding SUD, and the existence of suicide attempts seems to be a determining factor [44].

Until now, the combined measurement of neurocognition, circadian rhythmicity and molecular genetics in the diagnostic entities that we propose to study has never been addressed. This research could allow us to elucidate the presence of endogenous and exogenous explanatory factors and, if possible, with a predictive capacity of clinical interest.

### 1.4. Personality Characteristics

There is a large amount of evidence that indicates that certain personality characteristics, evaluated with multiple questionnaires that underlie different theoretical models, would be risk factors for the development of addictive behaviors and psychopathological disorders, also related to cognitive performance, clinical course and adherence to treatment. The existence of a vulnerability endophenotype is currently pointed out to develop an association between SUD and high Neuroticism-Anxiety and Impulsivity-Sensation-Seeking. If the disorder is developed, greater severity of addiction, craving and relapse are related to high scores of both personality traits [7]. In addition, treatment dropout occurs to a greater extent in patients with low scores in the Reward Dependency and Persistence traits [45].

Despite the heterogeneity of designs of previous studies with DD, we can point out that in male patients, there is a specific personality pattern, where SUD men tend to present with high Neuroticism-Anxiety and Impulsivity-Sensation-Seeking characteristics, and SMI men tend to show high Avoidance and low Persistence, regardless of whether SMI involves schizophrenia or major depression [46]. In dual depression, low Activity scores (ZKPQ) are specifically observed, being more evident with an early age at onset of SUD [7]. Low Activity is a feature present in the evening typology [47], and although it must be further analyzed, it could configure the personality endophenotype of dual depression and be related to polymorphisms of the *PER2* clock genes and *PER3*. On the other hand, in dual schizophrenia, low scores in Sociability are specifically observed [48], although this finding should be replicated in future studies with a greater number of patients and control of variables.

The implementation of therapeutic interventions aimed at the management of extreme personality traits has been more effective in individuals at high-risk of developing SUD than in classical cognitive or motivational therapies [49]. Similarly, in SUD patients, personality traits are beginning to be considered as a clinical marker, suggesting their usefulness in personalized treatments [50]. Our research aims to expand existing knowledge to DD, also linking it with clinical aspects of circadian rhythmicity and neurocognition to configure relevant and useful information in the therapeutic approach during the early remission phase. We consider the psychobiological-based personality questionnaires the most sensitive for this purpose, so we have been using the revised Cloninger Temperament and Character Inventory (TCI-R) and the Zuckerman-Kuhlman Personality Questionnaire (ZKPQ) based on the model of personality of the five alternative factors.

### 1.5. Chronobiological Therapeutic Approach to SUD and DD

The establishment of habits with regular sleep–wake schedules, meals and daily activities is very beneficial to maintain health and essential to recover it. In addition, these should be synchronized to the light–dark cycle with a morning pattern phase in which the contrast between light of day and night darkness is enhanced. It has been observed that the stability of habits is a protective factor for the development of mood disorders and to prevent relapses if they occur [12,51]. This therapeutic approach is based on fundamentals of chronobiology, although its implementation has been called “social rhythm therapy”, and it has been applied with good results in patients with bipolar disorder (see [51] for a review). The guidelines recommending stable habits and synchronization to the solar cycle are usually successfully incorporated into the withdrawal treatment of SUD, especially in the residential regime, regardless of the therapeutic approach [19,22]. However, investigation of the therapeutic effects of the establishment of habits in humans is scarce; information on compliance by outpatients and longitudinal efficacy data have not been collected in any case.

Because light is the main synchronizer of the human circadian clock, exposure to light has been proposed as a significant element to be incorporated into “social rhythm therapy” [51], whether natural or artificial light and preferably in the early hours of the morning [52]. Light has serotoninergic and melatoninergic agonist effects, which underlie the therapeutic actions explored so far.

Exposure to bright artificial light, ideally white full-spectrum light, is necessary when exposure to natural (solar) light is insufficient or not available, and it is the most frequent option in studies that have addressed the efficacy of light therapy. This has shown efficacy in reducing depressive symptoms in both seasonal and non-seasonal depression [53], as well as in insomnia and circadian sleep disorders (see [54] for a meta-analysis) at an intensity of between 2500 and 10,000 lux without differing greatly in the results for depression treatment [55]. Although exposure to light in the treatment of non-seasonal depression is effective in monotherapy regardless of sex, showing a faster response than that of antidepressants, some studies indicate greater symptomatic improvement in combination with antidepressant drugs (i.e., fluoxetine) [56,57]. The most common exposure periods are between 7 and 14 days, although it is suggested as ideal to maintain the treatment for between two and five weeks for non-seasonal depression [55]. A recent meta-analysis [58] concluded that bright white light, starting at 1000 lux, also improves daytime alertness and cognitive performance.

The study of exposure to natural light has provided data of interest, especially in the field of depression. Wirz-Justice et al. [52] observed that patients with seasonal depression responded to an hour of walking exposed to the light in winter (Switzerland) after only one week and also responded better than to exposure to artificial light of 2800 lux. The hospitalization time in patients with non-seasonal depression decreases if spaces are better illuminated, which has been observed in Mediterranean latitudes regardless of the season of the year [59], as well as further north in Holland [60]. In adults of not very advanced age, it promotes and adjusts the secretion phase of nocturnal melatonin [61], which in turn can correct abnormal functional patterns in the dopaminergic reinforcement system [62]. The intensity of natural light reaches therapeutic values without a problem, since it is estimated to be 3000 lux on a cloudy day, 10,000 lux on a normal day (approximate intensity of 45 min after sunrise) and 50,000 lux on a sunny day [61] (https://www.scribd.com/document/359698224/LightLevels-outdoor-indoor-es, accessed on 21 March 2022).

The rhythmic restoration difficulties observed in SUD patients [21] and dual depressive patients [22,63] under treatment, especially due to a worse quality of the daytime period, can benefit from incorporating adjunctive treatment of hourly habits and light exposure. This promotes the improvement of daytime activation, with its consequent benefits in the cognitive and affective state, and can reduce depressive symptomatology and the dysphoria of the withdrawal process in the case of dual patients. Natural light in our latitude (Barcelona, 41°38N) is suitable for this intervention, even in the shortest photoperiod months of the year [64].

Our project aims to obtain a clinical endophenotype of the differential vulnerability of DD, focusing on the two most common SMIs comorbid to SUD (major depression and schizophrenia) in clinical practice. For this purpose, a selection of measurements (circadian rhythm, genetic polymorphisms, neurocognition and personality traits) was included that may result in adherence and clinical course markers (with an emphasis on relapses) (Study 1). We also evaluated the efficacy of incorporating regular habits and exposure to natural light as an adjunctive therapy to the clinical management of patients with SUD and dual depression who show partial response to treatment compared to patients with similar characteristics who received the usual treatment (Study 2). Both studies represent a novel approach that may result in a significant advance in this field of knowledge.

## 2. Hypothesis and Objectives

### 2.1. Initial Hypothesis

1. Patients with DD will show worse social, clinical and quality of life characteristics, the SMI group being in an intermediate position and the SUD group showing the best profile in the majority of variables. Sex will influence this characterization, with worse clinical profiles in women.
2. Alterations of the circadian rhythmicity of peripheral temperature (lower amplitude and stability, phase delay, low percentage of rhythm, lower index of circadian function), with a predominance of endogenous deterioration, will be more present in patients with DD and SMI than in SUD patients, being related to the severity of the addiction, concomitant symptomatology, current age of patients and age at onset of the disorder.
3. Polymorphisms in the proposed candidate genes will contribute to prediction of clinical (MAOA), circadian (PER2 and PER3) and functioning profiles in neuropsychological tests (BDNF, MAOA) in patients.
4. Cognitive performance in neuropsychological tasks (attention, short and long-term verbal memory, working memory, cognitive flexibility and executive functions) will affect both SUD and DD patients compared to normative data. These will be modulated by sex and other variables, such as age, age at onset of the disorder, suicide attempts, tobacco use, main dependence substance, comorbid mental disorder, etc. The cognitive skills of patients with DD will be better than those of patients who only suffer from SMI (intradisorder comparisons).
5. The personality pattern of the SUD and DD patients will be characterized by the endophenotype of high Impulsivity-Sensation Seeking and Anxiety-Neuroticism, a pattern that will be configured with the additional feature of low Activity in dual depression and low Sociability in dual schizophrenia. This will differ between genders, with high Impulsivity-Sensation Seeking in men and high Anxiety-Neuroticism in women.
6. The deterioration of circadian rhythmic activity and cognitive performance will be related to a lower adherence to treatment and a higher rate of relapse, evaluated during a year of follow-up. In both cases, comorbidity will worsen the success of the treatment, with a greater impact on dual schizophrenia than on dual depression.
7. Chronobiological intervention in SUD and dual depression will improve rhythmic expression (increase in amplitude and interdaily stability of peripheral temperature). Benefits will be obtained in diurnal activation, mood and quality of life related to health and adherence to treatment in the early remission phase for both sexes. This will be observed in both diagnostic groups, with greater magnitude in the dual depression group and with respect to the control groups that follow the usual treatment in spite of criteria compliance. The positive data of the intervention will indicate the greater involvement and difficulty of restoring the S process than the C process (Borbély model) and will be associated with the presence of PER2 polymorphisms (rs934945) and worse cognitive performance prior to the intervention.

### 2.2. General Objectives

1) To study in patients with DD (major depression and comorbid schizophrenia) compared to SUD-only patients and SMI-only (major depression and schizophrenia) patients, as well as their clinical characteristics, circadian rhythmic functioning, presence of genetic polymorphisms, neuropsychological performance and personality characteristics in order to obtain a possible clinically cost-effective endophenotype of differential vulnerability for the diagnostic entities considered.
2) To establish clinical markers of treatment adherence, prognosis of evolution and risk of relapse (one year follow-up) that can be considered in the therapeutic approach.
3) To evaluate the benefit of incorporating regularly scheduled habits and exposure to natural light in the treatment of SUD and dual patients who exhibit difficulties regaining circadian rhythmicity after three months of treatment.

### 2.3. Specific Objectives

For study 1:

1. To describe the presence of genetic polymorphisms and the characteristics of circadian rhythmicity, neurocognitive performance and personality traits in patients with DD (comorbid schizophrenia and major depression) compared to patients diagnosed with SMI only and with SUD only. The registration of the endogenous and environmental components underlying circadian rhythmicity, the selection of polymorphisms of circadian genes and related to cognition, as well as the exhaustive consideration of cognitive performance and personality characteristics facilitates a novel and robust approach.
2. To describe the differential aspects in circadian rhythmicity, genetic polymorphisms, neurocognitive performance, personality traits and health-related quality of life in patients with DD according to comorbid SMI (major depressive disorder and schizophrenia).
3. To determine the presence of the variables most affected or with greater specific weight among those evaluated according to the characteristics of the SUD (age at onset, severity, etc.) and the comorbid diagnosis (major depressive disorder and schizophrenia). The influence of sex will be explored in the case of SUD and dual depression.
4. To explore relationships between clinical variables and the presence of rhythmic and/or cognitive deterioration, as well as genetic polymorphisms and extreme personality traits in the DD, SUD and SMI groups. These will be studied in SUD and dual depression conditions according to sex. This information will lead us to consider possible indicators/markers of vulnerability to be considered in clinical evaluation or treatment for an individualized approach.
5. To elucidate among the set of studied variables possible single or combined indicators of treatment adherence, as well as prognosis of evolution and relapses in SUD and DD to be used in future treatment and/or prevention for these patients.

For study 2:

1. To evaluate the benefits of adjuvant chronobiological therapy (regular habits in the schedule and exposure to natural light) for two months in abstinent patients with SUD and dual depression who show rhythmic restoration difficulties at three months of treatment.
2. To explore possible predictive variables of response to the efficacy of chronobiological therapy (genetic polymorphisms, cognitive performance, personality characteristics) in SUD and dual depressive patients of both sexes.
3. To compare the differential effects of chronobiological therapy with respect to usual treatment according to diagnosis (SUD and dual depression) and sex in terms of time and magnitude of the response in circadian rhythmicity (peripheral temperature and activity), clinical symptomatology and health-related quality of life.
4. To study the evolution of the chronobiological intervention of the SUD and dual depressive patients of both sexes with a one-year follow-up, also comparing the evolution of patients with the same diagnosis and similar clinical characteristics under usual treatment.

## 3. Materials and Methods

### 3.1. Participants

**Figure 1:**
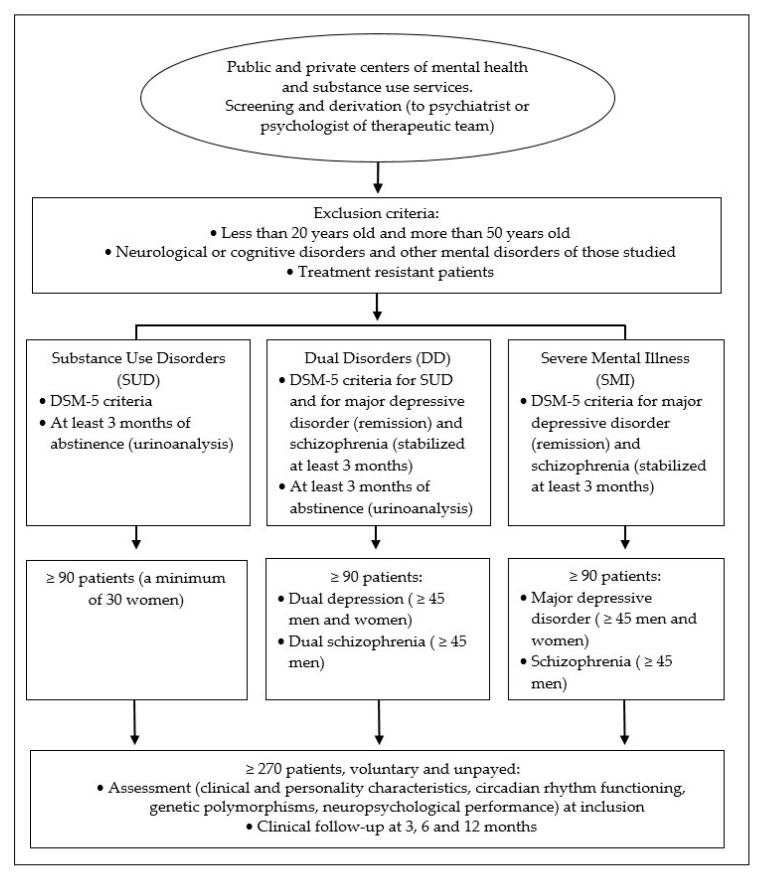
Inclusion and exclusion criteria of the groups of patients participating in Study 1.

For study 1, patients between aged 20 and 50 who presented only SUD, DD (dual depression or dual schizophrenia) and only SMI (major depressive disorder and schizophrenia) according to the Diagnostic and Statistical Manual of Mental Disorders (DSM-5) criteria [65]. A minimum of 90 patients will be included in each group, half of them diagnosed with major depressive disorder and schizophrenia in the DD and SMI groups. In the SUD group a minimum of 30 women will be included, whereas in the dual depression group a minimum of 20 women will be added to the sample of 45 men. Likewise, associated with the inclusion criteria of the second study, 45 DD patients with dual depression is the minimum, but we should probably include about 10 to 15 more. In all cases, the inclusion of nicotine and caffeine consumers will be allowed. Participation in the study will be voluntary and unpaid, after signing of informed consent by the patient or his legal tutors. See Figure 1.

For study 2, outpatients with a diagnosis of SUD and dual depression with a partial restoration of circadian rhythmicity will be selected. At least 30 patients will be included in each group, and ideally, 50% will be women. This will be determined based on the inclusion criteria of peripheral temperature values of SUD patients obtained by Capella et al. [21], which can be considered normative due to the high number of patients studied. Candidates will be those with an L10 (average 10 h with a minimum value) greater than 33 °C, an amplitude of less than 0.80 and an interdaily stability of rhythm of less than 0.5 (range 0–1). In addition, the daily distribution of activity and its exposure to light will be assessed not due to selection criteria but to individually emphasize the implementation of scheduled habits. The exclusion criteria will be: (a) presence of ocular pathologies (retinopathies, cataracts, etc.); (b) skin problems and/or eye sensitivity and photophobia; (c) obesity and metabolic syndrome; (d) practice of daily or weekly intense physical exercise; and (e) treatment with drugs known to modify circadian rhythmicity or produce sedative effects (i.e., agomelatine, antipsychotics, mood stabilizers and hypnotics). Patients will be alternately assigned to the condition of chronobiological approach adjuvant to the treatment of regular hour habits and exposure to light or to the usual treatment (control) with only the evaluations.

Sample size. Starting with the population of patients from the referring centers, with adherence to treatment during the inclusion period, the sample should be 239 patients (with a 95% confidence level and a 5% margin of error), compared to the 270 proposed in study 1. This would be an adequate sample size for the subsequent contrasts to be analyzed. For study 2, there are no data on patients with rhythmic alteration in SUD or dual depression, but the sample of our study exceeds that of all previous work on treatment with natural light in other more prevalent pathologies.

### 3.2. Instruments

Clinical evaluation. Sociodemographic data, family and personal psychiatric history, presence and characteristics of current diagnoses will be collected, along with age at onset, pharmacological treatments, tobacco consumption (if so, the Fagerström dependence questionnaire will be applied), caffeine consumption, assistance framework and treatment program, withdrawal time, relapses and previous suicide attempts, number of hospital admissions and duration and presence/absence of psychosocial problems. The health-related quality of life assessment will also be incorporated (SF-36 questionnaire) [66].

To rule out that the comorbid mental disorder in DD is secondary, the Spanish version of the Psychiatric Research Interview for Substance and Mental Disorders for DSM-5 (PRISM-5) [67] will be used. The severity of the SUD will be assessed using the Drug Abuse Screening Test (DAST-20) [68], the intensity of the depressive symptomatology using the Hamilton depression scale (HDRS) [69], the psychotic symptomatology with the PANSS scale [70] and suicidal risk using the Plutchik scale [71], all of them the Spanish version.

The evolution will be followed at 3, 6 and 12 months of inclusion in the study (application in pdf with closed fields that can be filled in on the computer), preferably through the contact professional of the center of origin or by the team’s researchers in a hetero-assessed manner, with the patient and consulting their medical history.

Circadian rhythmicity pattern. A Kronowise KW6^®^ ambulatory device (Kronohealth SL, University of Murcia, Spain) will be used to objectively record circadian rhythmicity. This integrates the measurement of peripheral/distal skin temperature, activity level by means of a 3-axis accelerometer and body position, as well as four light channels (average and peak of visible light, blue or circadian light that mimics the spectrum of melanopsin of retinal ganglion cells and infrared radiation). This integrated register allows for collection of variables that influence circadian rhythmicity (body position, physical activity, exposure to light, sleep and food schedules, etc.) in an objective and validated way [20,72]. An easy-to-use programming and reading-analysis software (Circadianware^TM^, Kronohealth, Murcia, Spain) has been developed and provides a complete circadian report. The device consists of a clock system placed around the non-dominant wrist, with a micro-USB connection for programming, data download and battery charging. Patients in study 1 should wear the device for 2 days, and those in study 2 will be monitored continuously during the first two weeks and at weeks 4 and 8, except in moments of personal hygiene, during which they can be removed (indicated in the “event” option of the Kronowise KW6^®^). In addition to the usual sleep schedule, on the days of registration, bedtime and waking up times will be considered by the event marker available in the device. The use of ambulatory instruments in mental health represents a significant progress both in assessment and, as recently evidenced, in therapeutic management [73,74].

Self-assessed information on circadian typology will also be collected using the Composite Morningness Scale (CSM) [75], mood rhythmicity pattern (MrhI) [76] and seasonal variations (SPAQ) [77], in all cases with validated instruments in the Spanish population. For study 2, patients will fill in eight unipolar visual analogue scales [78] daily after natural light exposition and during the two months of the intervention in order to assess subjective activation and mood status.

Genetic determinations. A 2 mL sample of saliva (Salivette Oragene-DNA (OG-500)) will be taken after 30 min of not having had any liquid or solid intake, including chewing gums or smoking. The saliva samples will be stored at room temperature in a light-preserved place until processed. For the extraction of genomic DNA, an aliquot of saliva will be used, following the manufacturer’s protocol (Oragene). A Qubit fluorometer (Invitrogen, Carlsbad, CA) will be used to determine DNA concentrations. The DNA samples will be adjusted to a final concentration of 10 ng/ul in TE-4 (10 mM Tris-HCl, 0.1 mM EDTA, pH 7.5) and stored at 4 °C until use. Molecular genetics analyses will be performed using previously validated real-time PCR-based methodologies, using Taqman probes for the PER2 polymorphisms (rs934945), PER3 (rs2640909), BDNF rs6265 (Val66Met), APOE rs429358 and rs7412 (E2, E3 and E4). The VNTR in MAOA will be genotyped by analyzing the amplified PCR fragments and run on agarose gel electrophoresis.

Neuropsychological evaluation. The battery of standardized neuropsychological tests is based on the evaluation of functions that may be affected in our sample. A measurement is avoided based on aspects such as reaction time or fine motor skills, since these may be influenced by the effects of the various psychopharmacological treatments used by patients, especially those with DD. The functions assessed and the tests applied are the following:

(a) Cubes (manipulative IQ) and Vocabulary (verbal IQ) from the WAIS-III [79].
(b) Attention. Block of direct digits from the WAIS-III Digit test [79].
(c) Short and long-term verbal declarative memory will be evaluated by the Rey’s Verbal Auditory Learning Test (RAVLT).
(d) Executive functions. Trail Making Test (Part A and B) and block of reverse digits from the WAIS-III Digits test for working memory; the Tower of Hanoi in its computerized version of 4 disks for planning and problem resolution; and the Wisconsin Card Sorting Task (WCST, Computerized Wisconsin Card Sort Task Version 4, 2003, Estevez-Gonzalez, Barcelona, Spain) for cognitive flexibility, concept creation, problem solving, inhibition and learning.

Finally, information will be collected with the Prefrontal Symptom Inventory (ISP) [80], consisting of 46 items that evaluate three factors: executive control problems (motivational, control and attention problems), social behavior problems and emotional control problems. This inventory has been validated in an adult clinical population with SUD, and convergence data with neuropsychological measures are also available [81].

Personality characteristics. Two personality questionnaires will be used that have been sensitive to numerous psychopathological disorders, including SUDs, and with positive data also in DD. Both are available in validated Spanish versions with population norms:

(a) Revised Temperament and Character Inventory (TCI-R) [82], based on the 7-factor personality model, consisting of 240 items with a response format on a 5-point Likert scale (1: strongly disagree and 5: totally agree). This considers 4 dimensions of Temperament of greater constitutional weight (Sensation Seeking, Persistence, Reward Dependency and Avoidance of Danger) and 3 of Character determined by more acquired personality aspects (Cooperation, Determination and Transcendence).
(b) Zuckerman-Kuhlman Personality Questionnaire (ZKPQ) [83], based on the Five-Factor Alternative Model and composed of a total of 99 dichotomous items (true–false). The Spanish version will be used, for which there are normative data [84]. The ZKPQ measures the dimensions of Neuroticism-Anxiety (N-Anx), Aggression-Hostility (Agg-Host), Activity (Act), Sociability (Sy) and Impulsivity-Sensation Seeking (ImSS).

### 3.3. General Procedure

Study 1. Candidates to participate in the study will be added gradually, as they are derived by the psychiatrist or psychologist in the therapeutic team from the clinical centers, who will have briefly explained what the study consists of. The study objective will be explained to all patients at the first visit (inclusion), along with the measurements to be carried out and number of sessions involving their participation. Those who accept will sign an informed consent (tutors in the case of incapacitation) and will be evaluated in the patient’s usual care center, preferably in morning sessions (9:00 a.m.–2:00 p.m.).

**Figure 2:**
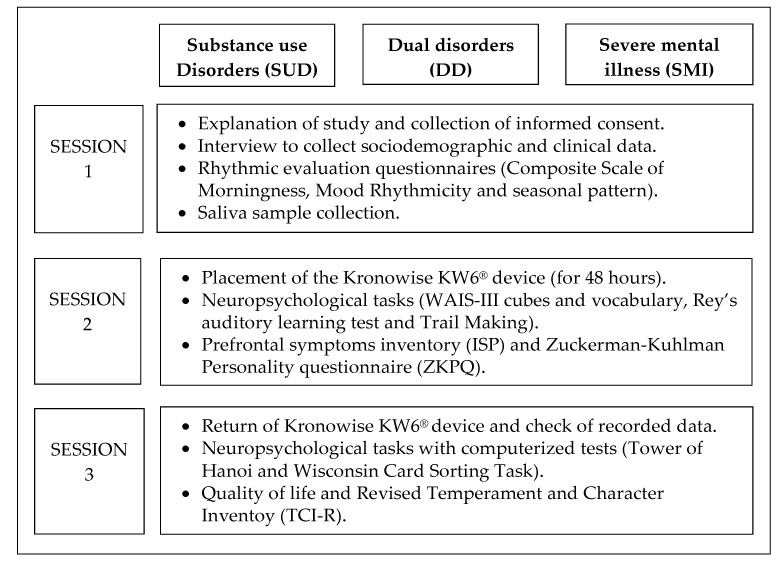
Breakdown of measurements for the sessions in study 1.

The collection of epidemiological and clinical information, as well as the application of the instruments, will be carried out by means of an individualized data collection booklet that will be completed either by the experimenter (hetero-application) or by the patient (self-application), depending on the case. Those tests that have a computerized version will be presented and answered on a computer. These are mostly performance tests that the subjects must answer individually. For the completion of the information, 3 individual sessions with the patient will be required, with a variable duration depending on the time it takes to respond but that will not exceed 2.5 h. The breakdown of sessions is presented in Figure 2.

A report of the results will be prepared for the clinical centers, and the return to the patient will be carried out by us or by a professional of the therapeutic team, according to the center’s decision.

Study 2. Outpatients of both sexes with a diagnosis of SUD and dual depression who have provided a circadian registry of peripheral temperature with a partial restoration of circadian rhythmicity will be proposed to participate in a chronobiological intervention complementary to the usual treatment. Those patients of both groups who agree to participate (given the link with the centers, a very high affirmative response is expected) will be assigned alternately to the treatment condition (habits and exposure to light) or to the control condition, with only routine treatment and ambulatory evaluations of rhythmic and subjective measures, as well as scheduled visits. The instructions of habits and exposure to natural light that will be stipulated for patients are presented in Box 1.

Box 1 **Instructions of habits and exposure to natural light stipulated for patients assigned to treatment condition in study 2. The text will be printed on a plasticized card that will be delivered on the day of inclusion to the patient so that they have it on hand and can check whenever they want.**

**Hourly habits:**

Get up before 08:00 a.m. and go to bed no later than 11:00 p.m.Sleep schedules will be regular, without changes on weekends or holidays.Have breakfast after getting up, lunch before 2:00 p.m. and dinner at about 9:00 p.m. After eating, you can rest, but please avoid falling asleep.Avoid bright blue light (electronic devices) one hour before going to sleep.


**Daily exposure to natural light:**
Go out every day between 9:00 and 11:00 a.m. for a one-hour walk. You can take short rest breaks, but it is better to keep moving and walking while exposed to daylight. Do not wear sunglasses or glasses that darken in contact with light. If weather conditions make it impossible to go outside, place yourself at the time of the walk next to a window with outside light for an hour while doing some activity (for example reading).

The intervention will last two months with the following visits and planned evaluations:

- Visit 1 (inclusion). Explanation of the study differentiated according to the assigned condition and collection of informed consent. Placement of the Kronowise KW6^®^ device that will be worn continuously until the next visit, emphasizing that the patient should register daily the time of getting up, having meals and going to bed. Specification of the rules to be followed in relation to hourly habits and exposure to natural light and delivery of the reminder card for treatment condition. Delivery and collection of the subjective assessment in 8 visual analogue scales (4 of activation and 4 of affective state) to be filled out by the patient each day until the next visit.
- Visit 2 (at 2 weeks of inclusion). Assessment of compliance with the chronobiological approach by the patient and possible adverse effects referred to us (open evaluation). Collection of the Kronowise KW6^®^ device and the visual analogue scales of these first 15 days and delivery of those that will be filled in the next 15 days. Application of the HAM-17 scale in patients with dual depression.
- Visit 3 (at 4 weeks of inclusion). Assessment of compliance with the chronobiological approach by the patient and possible adverse effects referred to us (open evaluation). Collection of visual analogue scale and delivery of those that must be completed until the next visit. Placement of the Kronowise KW6^®^ device that will be worn continuously for a week (it will be delivered on a routine visit to the center after the registration week). Application of the SF-36 to all patients and the HAM-17 scale in dual depression patients.
- Visit 4 (at 8 weeks of inclusion). Assessment of compliance with the chronobiological approach by the patient and possible adverse effects referred to us (open evaluation). Collection of the visual analogue scales and placement of the Kronowise KW6^®^ device that will be worn continuously for a week. Application of the SF-36 to all patients and the HAM-17 scale in dual depression patients.
- Visit 5 (at 9 weeks of inclusion). Assessment of compliance with the chronobiological approach by the patient. Collection of visual analogue scales and Kronowise KW6^®^ device. Recommendation to continue with the approach if the patient is satisfied and his sociolaboral activity allows it.

For the follow-ups at 3 (12 weeks), 6 and 12 months of inclusion, the same information will be collected from the patients in study 1 with a personal visit whenever possible.

Patients who agree to participate will sign a second informed consent that includes, for the intervention condition, the commitment to adhere to the recommendations of daily habits and daily exposure to natural light and to wear the Kronowise KW6^®^ device during the established periods, with specification of the control about their activity both night and day. Patients assigned to the control group will sign another simplified informed consent. Thanks to continuous ambulatory registry, adherence to both the schedules and the daily session of exposure to natural light will be controlled, along with the quantification of the intensity and type of light exposed and duration/intensity of physical exercise throughout the record. This prevents us from requiring the patient to register daily, even for shorter time periods of essential information, such as that collected with sleep diaries.

### 3.4. Statistical Analysis

The time series obtained from the Kronowise KW6^®^ records, using the Circadianware ™ software, will be subjected to a classic rhythmometric analysis with the cosinor method (maximum, minimum, mesor, amplitude, acrophase and % of the rhythm), as well as to an analysis non-parametric (interdaily stability, intraday variability, relative amplitude, etc.). The circadian function index (CFI) is calculated with the average of IS, IV and RA, ranging from 0 (absence of rhythm) to 1 (robust circadian rhythm).

The SNPStats program [85] will be used to calculate allelic and genotypic frequencies, as well as Hardy–Weinberg equilibrium analysis (with an *χ*^2^ test) to explore the association of SNP genotypes, with quantitative measures of the evaluated variables. A corrected linear regression model will be used for age and sex (when applicable). An approach based on the false discovery rate (FDR, q value) will be applied for the correction of multiple statistical tests, using the QVALUE program. The exploration of gene–gene and environment–gene interactions will be carried out with the MDR program and the multilocus genetic profile (MGP) method.

All independent and dependent variables collected in the study will be incorporated through a double entry system in a computer file after conversion of the data involving hours to the centesimal system. In the dependent variables for which population scales are available (i.e., percentiles), these will be considered or Z or T scores will be calculated as appropriate. Descriptive statistics and a complete correlational analysis will be calculated for the different variables considered. Subsequently, different covariance analyses (ANCOVA/MANCOVA) will be carried out for the rhythmic pattern, neuropsychological performance and personality factors, as dependent variables, introducing group (DD, SUD, SMI) as an independent variable and the age as covariate in all cases. The same analyses will be carried out for the SUD and dual depressive patients, incorporating sex as a factor. If the conditions for the analyses are not met, equivalent non-parametric tests will be used. The need to apply Bonferroni correction for multiple comparisons will also be assessed. The consideration of other independent variables to be included as covariates will be determined from the descriptive and correlational analyses (residential/outpatient treatment, age at onset of the disorder, months of abstinence, etc.). All analyses will also be performed comparing only the DD and SMI groups according to the type of mental disorder. For study 2, repeated measures analyses will be carried out with the different temporal measurements and with intervention (treatment and control), diagnosis (SUD and dual depression) and sex as factors, as well as contrasts between groups at each time considered. In all cases, the eta squared partial statistic (η_p_^2^) will be estimated to measure the effect sizes. Linear or logistic regression models will be carried out, if applicable, that include as predictive variables those present in the clinical history in relation to the measurements made in both studies and, of these, in relation to the information of the follow-ups. The analyses will be carried out with the SPSS/PC+ statistical package (SPSS Inc., Chicago, IL, USA), and the statistical tests will be considered bilaterally, with a type I error established at 5%.

### 3.5. Management and Collection of Research Data

The protocols for the two studies are in accordance with Spanish legislation (Biomedical Research Law, BOE 4 July 2007, Research on data collection in humans). Our research adheres to the ethical standards of the Declaration of Helsinki [86] and of research in chronobiology [87]. Furthermore, the procedures will be carried out in accordance with international recommendations in the field of ethics of human genetic studies [88]. Participation in the studies does not imply risks for patients, as there are no invasive registries or interventions with known risky side effects.

Data collected from the research group for the Project will be digitized and stored on the University’s Microsoft OneDrive for Business. The Microsoft Agreement includes Terms and Conditions that are compliant with EU Data Protection Law and the National Bioethics Committee rules and regulations. Only researchers working for the Project will have access to the data, using their username and passwords to access the files.

## 4. Discussion

The results of this project can contribute significantly to the knowledge about patients with SUD and DD both at a basic or theoretical level and for intervention and relapse prevention. All the results obtained from the two studies, but especially the relationships between the different variables evaluated (clinical characteristics, genetic polymorphisms, circadian rhythmicity, neurocognitive functioning and personality traits), will represent, in most cases, the first data obtained in the field both at national and international level. The adjuvant chronobiological therapy intervention will be the first study to be carried out with this type of patients and with an objective outpatient evaluation that provides us with information on patient compliance and changes in circadian rhythmicity. The consideration of sex in SUD and dual depression is a pending task due to the non-proportional prevalence of cases in the clinic, which we will carry out in a novel way both at the level of characterization and intervention.

Currently, both SUD and DD are disorders of high prevalence in drug dependence and mental health care worldwide, with difficulties in therapeutic management and with high relapse rates. The search for biomarkers or endophenotypes likely to improve adherence and response to treatment is a relevant pending issue. Furthermore, exploring the option of improving the approach by means of chronobiological strategies will be a pioneering contribution that can be transferred to the clinical setting, as well as being disseminated at a social and media level.

Likewise, our research can provide recommendations in relation to considering aspects that benefit the evaluation and diagnosis protocols and/or the convenience of incorporating some strategies in the therapeutic management of patients (i.e., cognitive rehabilitation), including the sex dimension. We also hope to delimit some cost-effective markers of adherence, prognosis and risk of relapse, as well as the existence of rhythmic characteristics in combination with modifiable genetic polymorphisms in case they are found to be altered. With all this, the present research will result in a contribution in line with the general objectives proposed by the WHO for mental illnesses [89] and specifically in the clinical management of patients with addiction and DD [90]. Finally, the proposed intervention, if it proves to be useful, will be the first work carried out worldwide with a potential clinical sample (SUD and dual depression) that could unquestionably benefit from it.

From a social-impact point of view, since the Nobel medicine/physiology award of 2017 (Jeffrey C. Hall, Michael Rosbash and Michael W. Young) for research on the molecular mechanisms that control circadian rhythms, media interest in chronobiology has notably increased. DD is a health issue to which the media are beginning to be sensitive, especially promoted by the debate on the legal situation of cannabis and the coexistence of psychotic disorders among its consumers. The media have also recently echoed the impact that the coronavirus pandemic has generated on consumption patterns and increases in SUD. The dissemination of the results of study 1 will make visible the high presence of psychiatric comorbidity in SUD, as well as its personal and social impact, together with recommendations based on useful markers in clinical management. The data from the chronobiological intervention, if positive results are obtained, will undoubtedly be likely to be disseminated not only in our country but internationally.
